# Taking hold of hand trauma in KwaZulu-Natal, South Africa

**DOI:** 10.4314/ahs.v21i4.35

**Published:** 2021-12

**Authors:** Jenousha Naidoo, Pragashnie Govender, Deshini Naidoo

**Affiliations:** 1 Department of Health; 2 University of KwaZulu Natal (Westville campus), Occupational Therapy

**Keywords:** Occupational therapy, traumatic hand injuries, hand rehabilitation

## Abstract

**Background:**

Trauma in KwaZulu-Natal province in South Africa constitutes at least 17.8% of overall emergency cases, with hand trauma being common.

**Aim:**

Based on these statistics, the authors of this study aimed to identify and describe the most common traumatic hand injuries managed in the province including current trends and intervention practices of occupational therapists to inform future intervention.

**Methods:**

Using a mixed-method convergent parallel design, 41 therapists completed an online survey, and 12 therapists participated in two focus group discussions. Survey responses were analysed using descriptive statistics, and the audio-recorded and transcribed focus group discussions were analysed deductively using thematic analysis.

**Findings:**

Flexor tendon injuries (88%), extensor tendon injuries (73%), fractures (83%) and combined hand injuries (73%) were the most common injuries noted. Sufficient theoretical knowledge (95%), clinical judgement (93%), available resources (88%), relevant practical experience (83%) and surgeon hand therapy protocols (88%) were identified as essential in managing traumatic hand injuries. Challenges included having limited resources, late referrals and poor communication hindering multidisciplinary practice.

**Conclusion:**

Therapists face challenges in managing traumatic hand injuries, which inhibits optimal intervention planning. These factors may inevitably negatively influence outcomes achieved through occupational therapy for this group of patients.

## Introduction

The human hand is a highly complex anatomical structure, which allows humans to have tactile feedback, fine-motor precision, dexterity and engagement in activities of daily living^1^,^2^. Hand injuries are the most common of all bodily injuries, with an increasing number of traumatic hand injuries (THI) presenting to emergency services globally^3^. In the province of KwaZulu-Natal (KZN) in South Africa (SA), trauma is extensive, constituting at least 17.8% of overall emergency cases^4^. Anecdotal evidence suggests that hand trauma is common amongst the patients managed by occupational therapists (OTs); however, there is a lack of contextually relevant evidence to guide practice. A hand injury causes pain, trauma and subsequent physical, psychosocial and social consequences, which can lead to either temporary or permanent decline in hand function^5^. Additionally, the financial burden resulting from a hand injury in terms of intervention, time off from work and possible job loss has a significant psychological influence on the person and affects overall recovery^6^. It is thus vital for healthcare professionals to manage a client holistically, with due cognisance of the contributing elements and not just the biomechanical and physical factors.

Gupta and colleagues^7^ postulate that there are only a few other injuries that compete with the injured hand in complexity. Hand injuries require a multidisciplinary team (MDT) approach, which includes integrated planning between medical and rehabilitation specialists to attain optimal recovery and function^7^. Moreover, the management of hand injuries is costly, and these costs may increase if the client develops complications resulting from incorrect intervention^10^. 11, 12.

Hand therapy is an area of special interest within occupational therapy (OT) practice that is essential for the restoration of hand function^8^. This paper seeks to describe the current trends and intervention practices of OTs working in the field of hand rehabilitation, their perceptions and experiences related to their preparedness for practice in the area of hand rehabilitation of THI. Essential considerations in the management of traumatic hand injuries to inform future intervention provided by OTs is considered.

## Literature review

### Traumatic Hand Injuries

Both international and local literature highlights that THI's vary in severity and complexity and may severely affect a person's ability to perform daily activities 7–24. International literature highlights that THI's vary in severity and complexity and may severely affect a person's ability to perform daily activities as well as their life roles, either temporarily or permanently 7, 23,24.

In high-income countries the United Kingdom, 20 per cent of patients attending emergency services included hand injuries, resulting in 1.36 million hand injuries seen in a year, of which 271 000 require surgical intervention^13^. Angermann and colleagues^14^ found that an average of 14 per cent to 30 per cent of all patients treated in Danish emergency care included hand injuries. Similarly, in a European study, Clark and colleagues^15^ revealed that hand injuries were accountable for 10 per cent of all emergency department visits and 20 per cent of all injuries treated. In lower-to-middle income countries (LMIC), the prevalence of THI is lower with a study in Uganda, reporting the burden of hand injuries to be 5.5 per cent in trauma services^6^. However, Stewart and colleagues in their study^17^ indicated that THI accounted for one-third of all traumatic injuries treated at South African public sector hospitals.

The spectrum of THI is vast, ranging from minor injuries and fractures to more complex injuries requiring nerve, tendon and artery repair^16^. In terms prevalence of THI in India, data reveals that closed fractures were the most common injuries, followed closely by soft tissue injuries of the hand^7^. Similarly, a Dutch study^14^ found that the most common injury in their sample were fractures (42%), followed by tendon injuries (29%). This differs from the results in a study in Uganda which found that hand lacerations were the most frequent injuries as seen in 32 % of their patients, with fractures only contributing a mere 16 % of the sample^6^. The variability of these statistics indicates that the prevalence of THI is variable and may present as unique to different locations hence the need for additional literature around THI from specific contexts.

### Occupational Therapy in Hand Rehabilitation

OTs in the field of hand rehabilitation are typically involved in the management of orthopaedic conditions such as fractures, lacerations, amputations, burns, tendon and nerve repair, as well as acquired conditions such as tendonitis, arthritis, and carpal tunnel syndrome^25^. OT intervention addresses the biomechanical complications of a hand injury as well as the functional and the psychological implications^25^. Therapy focusses on facilitating the functional recovery of the affected hand and promoting the return to premorbid functioning^26^. Hand rehabilitation by an OT consists of an initial evaluation to determine client-centred intervention goals, preparatory methods and purposeful activities, and possible provision of splints and adaptive devices^25^. Physiological factors addressed in hand therapy include pain, stiffness, oedema, tissue healing and scarring and these may be managed using splinting, manual techniques, and individually designed activities, amidst others^27^,^28^. In SA, there is currently limited information on the role of OT in THI, with a few recent studies that considered general hand therapy practice in the country^18^–^22^. Stormbroek and Buchanan's^19^ study explored 104 novice OTs experience of providing hand rehabilitation intervention. They discovered that these therapists managed an average of 20 clients per month. Of these, central nervous system conditions (91.3%), bone and joint (72.8%) and arthritic conditions (72.4%) were most frequently seen. In KZN, there has been only one recent study that considered the profile and management of the firework-injured hand^21^. There remains a paucity of literature on the current intervention practices for management of THI's by OTs in KZN.

## Methods

A mixed-method convergent parallel design (survey and focus groups)^29^ was used to gain insight into the burden of THI and current practices from the perspective of therapists. Purposive homogenous sampling^30^ was used to select participants who were OTs employed in KZN, registered with the Health Professions Council of South Africa and who had been exposed to hand rehabilitation within the prior five years. An invitation to participate in the survey was sent via email. The survey was open for eight weeks with one email reminder in this period. Participants were contacted via email to request participation in the focus groups. Two focus groups of approximately 60 minutes duration were conducted in the eThekwini and uMgungundlovu districts of KZN, each with six participants (n=12). Six to eight participants was a large enough group to attain diverse information 34.

A survey was developed using available information from the search of the literature and aligned to the study aim. The survey was based on Knowledge, Attitudes and Practice (KAP) to describe OTs knowledge, attitude and practice with regards to THI. According to Vandamme^31^, there is no clear methodology to develop a KAP questionnaire with different studies using different formats. The questionnaire was divided into six sections, namely: consent, biographical data, THI, knowledge of THI (how do they seek knowledge), attitude towards THI (how do they feel) and practice of THI (what do they do). The questionnaire was administered electronically and consisted of all closed-ended questions with the option of including comments if required. Questions for the focus group centred around the most common THI seen by therapists in their institutions, their perceived competency in managing THI, preparedness for practice from training and experience, what they considered essential for therapists in managing THI as well as barriers and enablers to intervention for THI in their current contexts.

The survey was exposed to five individuals that were active in the field of hand rehabilitation in the province. Ambiguous questions were reworded and restructured, and the order of items were realigned to maintain coherence. Face validity of the survey was thus achieved^32^. Trustworthiness of the study was ensured by peer debriefing, an audit trail and reduction of researcher bias^33^ Descriptive statistics were utilised to analyse the data from the surveys using frequencies and percentages. The audio recordings from the focus groups were transcribed verbatim and analysed using deductive thematic analysis^34^. Following the individual analysis, data were merged and interpreted jointly and is presented in a combined narrative.

Ethical approval was obtained from the Biomedical Research Ethics Committee (BE203/16). The ethical principles taken into consideration were the participant's right to withdraw, anonymity, informed consent, beneficence and non-maleficence. Principles of scientific honesty and integrity were adhered to in this study.

### Findings

#### Demographic Profile of Participants

A total of 41 therapists completed the survey, and 12 therapists participated in the focus group discussions. The majority of participants were within the 20–30 year age band, had up to ten years of clinical experience and holds a bachelor's degree in OT. There was representation from each level of the public health care system (Table I).

**Table I:** Demographic profile of participants

	*Characteristics*	*Survey (n=41)* *n (%)*	*Focus Groups* *(n=12)* *n (%)*
***Age***	20–30 years	26(63)	84(10)
	31–40 years	7(17)	1(8)
	41–50 years	8(20)	1(8)
***Experience***	1–5 years	19(46)	6(50)
	6–10 years	11 27)	5(42)
	11–20 years	4(10)	1(8)
	> 20 years	7(17)	0(0)
***Highest Level*** ***of Education***	Bachelor's degree	35(85)	11(92)
	Master's degree	4(10)	1(8)
	Postgraduate Diploma	2(5)	0(0)
***Facility***	District public hospital	13(31)	3(25)
	Regional public hospital	11(27)	3(25)
	Private (Practice/Hospital/Rehabilitation Facility)	8(20)	0(0)
	Tertiary public hospital	6(15)	5(42)
	Community Health Care Centre	3(7)	1(8)
***Undergraduate*** ***Training***	University of KwaZulu-Natal	31(76)	11(92)
	University of Free State	4(10)	1(8)
	University of Cape Town	2(5)	0(0)
	University of the Witwatersrand	2(5)	0(0)
	University of Pretoria	1(2)	0(0)
	Sefako Makgatho Health Sciences University	1(2)	0(0)

#### Common Traumatic Hand Injuries in KwaZulu Natal (Table II)

**Table II:** Frequency of THI

THI Group and Subgroup	(n=41) n (%)
Amputations	19 (46)
Bites	12 (29)
Burns	20 (49)
Crush Injuries	18 (44)
Dislocations	11 (27)
Extensor Tendon Injuries	30 (73)
Flexor Tendon Injuries	36 (88)
Fractures	34 (83)
Lacerations	16 (39)
Peripheral Nerve Injuries	19 (46)
Combined Hand Injuries	30 (73)
Soft Tissue Injuries	17 (42)
Vascular Injuries	7 (17)

Flexor tendon injuries emerged as the most common hand injury managed by OTs in the last year of practice (88%, n=36), followed closely by fractures (83%, n=34). The third most common THI were extensor tendon injuries (73%, n=30) and combined hand injuries (73%, n=30). The therapists in the focus groups similarly expressed the opinion that flexor tendons, extensor tendons, nerve injuries and fractures were the most commonly seen in their daily practice. Vascular injuries were the least common THI (17%, n=7).

#### Current Trends and Practices of OTs in Hand Rehabilitation

The current practices of therapists, namely, response to referrals, assessment, intervention planning, intervention, and follow up, in the management of THI, were explored and are described in a combined narrative.

*Referral systems and response to referrals:* Patients with THI are mostly referred to OT as outpatients (83%, n=34), followed by referrals as an inpatient post-surgical management (68%, n=28). OTs also refer patients to other members of the MDT, these include physiotherapists (83%; n=34), orthopaedic surgeons (71%; n=29), orthotists (29%; n=12), and plastic surgeons (24%; n=10). Therapists in the focus groups raised the issues of inappropriate referrals, patients not being referred or late referrals. Moreover, therapists prefer not to refer to new graduate therapists at the base hospitals due to their perception that these therapists lack relevant knowledge and experience.

“*Sometimes we refer some tendon injuries postoperatively back to their base hospital and often at their base hospital there's community service therapist and then they not sure about the regime or like early complication signs and then when the patient comes back then you picking up contractures*” (AG)

The response time to the management of THI was also identified (Table III). Majority of the cases were referred within the same day to within one week. However, several injuries such as amputations and fractures were referred only four to six weeks following the injury resulting in delayed interventions.

**Table III:** Response to Referrals by Therapists for THI (n=41)

THI Group	Same Day n (%)	Within 1- week n (%)	2–3 weeks n (%)	4–6 weeks n (%)	At the advice of the medical practitioner n (%)
**Amputations**	15 (37)	16 (39)	2 (5)	7 (17)	1 (2)
**Bites**	15 (37)	24 (59)	1 (2)	1 (2)	0 (0)
**Burns**	19 (46)	18 (44)	0 (0)	3 (7)	1 (2)
**Crush injuries**	17 (42)	20 (49)	1 (2)	3 (7)	0 (0)
**Dislocations**	20 (49)	18 (37)	0 (0)	3 (7)	0 (0)
**Extensor tendon injuries**	20 (49)	17 (42)	1 (2)	3 (7)	0 (0)
**Flexor tendon injuries**	19 (46)	19 (46)	1 (2)	2 (5)	0 (0)
**Fractures**	23 (56)	12 (29)	1 (2)	5 (12)	0 (0)
**Lacerations**	18 (44)	17 (42)	2 (5)	4 (10)	0 (0)
**Peripheral nerve injuries**	19 (46)	18 (44)	0 (0)	4 (10)	0 (0)
**Combined hand injuries**	16 (39)	19 (44)	4 (10)	3 (7)	0 (0)
**Polytrauma**	19 (46)	19 (46)	1 (2)	2 (5)	0 (0)
**Soft tissue injuries**	18 (44)	19 (46)	0 (0)	4 (10)	0 (0)
**Vascular injuries**	21 (51)	16 (39)	0 (0)	4 (10)	0 (0)

*Assessment:* Majority of the therapists (73%; n=30) reported assessing patients during the initial visit. Informal assessments (56%; n=23) and a combination of standardised and informal assessments (39%; n=16) were noted. Standardised assessments used included grip strength with use of a dynamometer (42%; n=17), the Disabilities of the Arm, Shoulder and Hand (DASH) questionnaire (17%; n=7); the Semmes Weinstein monofilaments sensory test (12%; n=5) and the Purdue pegboard test (12%; n=5). Additional tests that were utilised included, the WASP (2%; n=1); the ninehole peg test (2%; n=1); Jebsen-Taylor Hand Function Test (2%; n=1), O'Connor dexterity test (2%; n=1), Minnesota Dexterity Test (2%; n=1) and use of a pinch gauge (2%; n=1).

*Intervention:* Within intervention planning, therapists indicated their preferred rationale for intervention by highlighting their type of reasoning used, as well as the factors that influenced their management of clients of THI. The therapists were provided with five treatment approaches, namely, (i) interactive reasoning (considering the impact the injury has on an individual's life), (ii) narrative reasoning (considering an individual's daily activities, habits and roles), (iii) pragmatic reasoning (considering factors such as reimbursement, documentation, resources, discharge environment, therapists knowledge and skill), (iv) conditional reasoning (considering premorbid functioning, current status, prognosis, social and physical environment as well as the co-operation of the individual), and (v) procedural reasoning (considering the individuals diagnosticaly related performance components and areas). Majority of the sample (80%, n=33) used conditional reasoning, 10% (n=4) used narrative and pragmatic reasoning, 7% (n=3) used procedural reasoning and 2% (n=1) used interactive reasoning.

**Figure 1:**
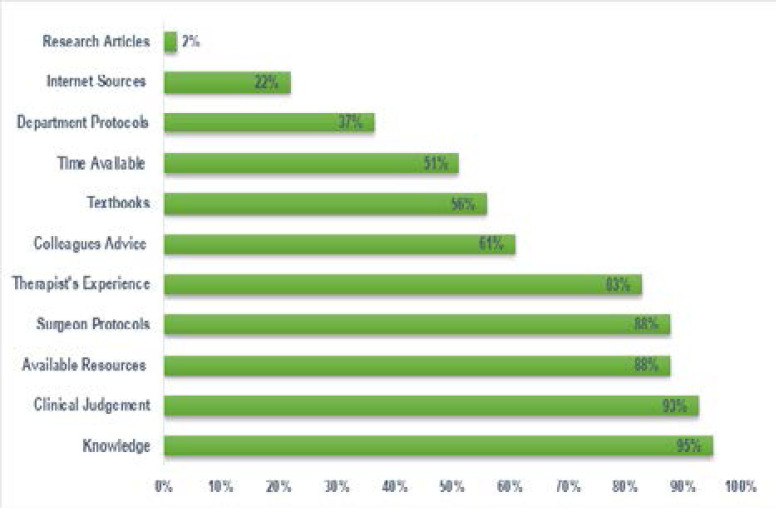
Considerations in Intervention Planning (n=41)

There were several factors that the therapists considered in intervention planning (Figure 1). Majority of the therapists indicated knowledge of the condition (95%; n=39) and their clinical judgement (93%; n=38) as key aspects of intervention planning. This was followed by available resources and surgeon protocols (88%; n=36), with 83% (n=34) considering their experience as a therapist with the condition. Other factors included advice from colleagues (61%; n= 25); textbooks (56%; n= 23); time available (51%; n=21) departmental protocols (37%; n=15); internet sources (22%; n=9) and research articles (2%; n=1). Additional factors that emerged from the focus groups included consideration of the patient's level of functioning, therapist's level of interest, stage of healing, phase of rehabilitation, pain levels of the patient and discharge planning.

“*...three components that serve as a blueprint for therapy: your stage of healing, anatomy and your safe positions of the hand. And then whenever that overlap is what you are going to do, based on your clinical reasoning*” (GR)

There were numerous elements outlined by therapists as being essential for the intervention of THI in practice. These included considered having adequate theoretical knowledge (88%; n=36), sufficient practical experience (88%; n=36) and having protocols, procedures and guidelines (88%; n=36). This was followed by having adequate training (78%, n=32) and adequate mentorship (71%, n=29).

Therapists in the focus groups highlighted assessment skills, knowledge of conditions, and extensive knowledge of anatomy and necessary splinting skills as additional essential elements in the management of a THI. Furthermore, therapists also acknowledged the need for adequate training, communication with the MDT, sufficient resources, adequate home programmes, patient compliance, and good relationship with the referring specialist.

“*I think a good relationship with the orthopaedic surgeons or doctors makes a big difference and I think that you can see it in those hospitals that have hand clinics where the OT's work together with the doctors, where the doctors are giving you information and you are discussing it openly*” (XM)

### Challenges experienced by OTs

A lack of resources was reported to be a significant obstacle (88%; n=36) of therapists' reporting that they base management of a THI on available resources. Therapists in the focus group raised the issue that intervention was limited due to the lack of resources. Contrarily, one therapist reported that you could do ‘amazing stuff’ without resources based on your clinical reasoning. The challenge with resources seems to be predominantly in the public sector, more so with the lower-level facilities such as community health centres, and district rather than tertiary institutions. Therapist's highlighted long waiting time for procurement and OT items not been that recognised as a priority as contributing factors to limited resources.

“*The orders just take so long [to arrive] that we run out of material before the next batch comes, and then we start turning patients away. And so we are very limited in providing the intervention that we could do*” (AN)

These contextual challenges, however, had a positive spin-off, as illustrated in this comment:

“*We go through stages where we recycle splint material .... You have to be creative and think out of the box*” (GR)

Patient adherence emerged as a significant challenge faced by the majority of the therapists in the focus groups. Poor patient adherence was attributed to language barriers, patients' defaulting on appointments and contextual difficulties such as poverty where the potential to obtain a disability grant reduces the patient's motivation to be adherent to therapy protocols.

“*...As soon as people start talking about temporary disability grants, I want to say no. But yet I understand that most of our people are indigent and they need the money, but the moment you throw that grant into the mix, you can say goodbye to that hand*.” (JB)

Communication and the relationship within the MDT emerged as one of the many challenges as it was found to be difficult when team members are not cooperative.

“*... it's difficult with communication with the MDT to speak to someone on the phone that thinks you giving instructions but you actually just trying to get a multidisciplinary approach for the patient...*” (NF)

## Discussion

This study aimed to highlight the OT perspective on the management of THI in KZN, SA. The most common THI highlighted in this study were fractures, flexor and extensor injuries and combined injuries. Gupta and colleagues^7^ in India, Angermann and team^14^ in Denmark, and Pietrobon^35^ in a previous SA study found similar trends with THI where fractures and tendon injuries were most common. The findings in this study differed from Van Stormbroek and Buchanan^19^ findings, where the most prevalent causes of hand injuries were central nervous system, bone and joint and arthritic conditions. The differences were attributed to these authors exploring general hand conditions rather than THI.

OT received THI referrals mainly as outpatients, which is in keeping with current hospital practice^36^. Referral to other members of the MDT included physiotherapists, orthopaedic surgeons, orthotists and plastic surgeons, which is in keeping with the MDT approach advocated by the British Society for Surgery of the Hand^35^. However, the OTs in this study raised the issue of inappropriate or late referrals, which hindered intervention, which was congruent with one other retrieved study^37^. There were variations in response time to the management of THI. From this study, it appears that most injuries were attended to on the same day, with most traumatic hand conditions being seen within one week. Similarly, an Australian study^38^, found that limited staff influenced the effective provision of hand therapy services. Early intervention of traumatic injuries in ideal situations can also improve patient outcomes^39^.

OTs also felt patient outcomes were dependent on the experience of the treating therapists and OTs at tertiary hospitals were reluctant to refer clients to the district level hospitals. This was primarily attributed to the fact that community service therapists, who predominantly staff the district hospitals, were perceived by the more experienced therapists as not being able to cope with the complexity of hand therapy intervention. Similarly, Naidoo and colleagues^26^ investigated the preparedness of final year students for practice and established that students appeared to feel confident to cope with basic practice but suggest that curricula be improved to ensure greater confidence and competence in hand therapy.

The OTs in this study used both informal and formal assessments with the dynamometer, Disabilities of the Arm, Shoulder and Hand questionnaire (DASH), the Semmes Weinstein monofilament sensory test and the Purdue pegboard test being the most common assessments. These findings are similar to De Klerk and colleagues^22^, which postulated that body structure and function were focused predominantly on body structure and function. However, OTs in the focus groups in this study, voiced that they consider the patients level of functioning when planning intervention, which may be a reference to their clinical reasoning and not assessment practices per se.

The management of hand injuries should consider a person holistically, taking into account their roles, needs and goals^39^. Conditional reasoning, with clinical judgement and knowledge of the condition, were found to be essential for intervention planning in this study. Protocols, procedures and guidelines, having adequate theoretical knowledge and having sufficient practical experience were considered necessary for the intervention of THI. Furthermore, the OTs primarily use clinical judgement and hand therapy protocols to guide their intervention approaches. A combination of these can be considered ideal; with relevant current protocols providing the outline of intervention and clinical judgement assisting with adapting the protocol to the patient's individual needs. There, however remains the challenge in ensuring that these protocols are current and evidence-based. Hand injuries are complex on their own, but the OT management of these injuries comes with its own set of challenges. Therapists identified having limited resources, patient compliance factors and dealing with the MDT as some of the challenges, they face. Limitations with resources in terms of equipment, consumables and staff compliment were raised as a significant concern for therapists as this negatively affects service delivery. South African therapists are faced with large caseloads, swift turnover of referrals, limited space and resources^22^. The patient's compliance is key to successful intervention. Adherence is affected by numerous factors such as the patient-practitioner language barrier, difficulty attending therapy due to financial constraints, the possibility of receiving a disability grant, as well as patient attitudes^22^, ^40^.

Therapists expressed communication to be the underlying problem with team care; as each department, be it orthopaedics, OT, physiotherapy or others, have their own set of procedures, and these may not necessarily be aligned with the others. Although the therapists in this study worked at institutions, which have orthopaedic clinics, there is a limited collaboration with the MDT, resulting in poor team management of the THI.

## Conclusion

This study was localised to KZN, used a purposive sample, and hence cannot be generalised to the broader OT population. Notwithstanding this, the issues around hand trauma and current practices provide valuable information that may be used for improved practice and training.
